# *TERT* Promoter Mutation C228T Increases Risk for Tumor Recurrence and Death in Head and Neck Cancer Patients

**DOI:** 10.3389/fonc.2020.01275

**Published:** 2020-07-28

**Authors:** Lidia Maria Rebolho Batista Arantes, Adriana Cruvinel-Carloni, Ana Carolina de Carvalho, Bruna Pereira Sorroche, André Lopes Carvalho, Cristovam Scapulatempo-Neto, Rui Manuel Reis

**Affiliations:** ^1^Molecular Oncology Research Center, Barretos Cancer Hospital, Barretos, Brazil; ^2^Department of Head and Neck Surgery, Barretos Cancer Hospital, Barretos, Brazil; ^3^Department of Pathology, Barretos Cancer Hospital, Barretos, Brazil; ^4^Pathology and Molecular Diagnostics Service, Diagnosticos da América-DASA, Barueri, Brazil; ^5^Life and Health Sciences Research Institute (ICVS), Medical School, University of Minho, Braga, Portugal; ^6^ICVS/3B's-PT Government Associate Laboratory, Braga, Guimarães, Portugal

**Keywords:** HNSCC, *TERT* promoter mutations C228T and C250T, prognostic biomarker, disease-free survival, overall survival

## Abstract

**Background:** Head and neck squamous cell carcinoma (HNSCC) is usually associated to tobacco and alcohol consumption. Increased telomerase activity has been consistently detected in 80–90% of malignant tumors, including HNSCC. Mutations within the promoter region of telomerase reverse transcriptase (*TERT*) that confer enhanced *TERT* promoter activity have been reported in two major hotspots, designated C228T and C250T.

**Objectives:** To evaluate *TERT* promoter mutations C228T and C250T in HNSCC patients from Brazil and correlate with patients' outcome.

**Materials and Methods:** Formalin-fixed paraffin-embedded tissues were obtained from 88 HNSCC patients and analyzed for *TERT* promoter mutations C228T and C250T by pyrosequencing.

**Results:** The overall prevalence of hotspot *TERT* mutations in HNSCC samples was of 27.3%, with 6.8% at locus C228T and 20.5% at C250T. The majority (92%) of mutated cases were located in oral cavity, mainly at the tongue. We observed that 94.4% of the patients harboring *TERT* promoter mutation C250T were alcohol consumers (*p* = 0.032) and 66.7% of the patients harboring *TERT* promoter mutation C228T were not alcohol consumers (*p* = 0.035). The presence of C228T mutation impacted patient outcome, with a significant decrease in disease-free survival (20.0 vs. 63.0%, *p* =0.017) and in overall survival (16.7 vs. 45.1%, *p* = 0.017).

**Conclusion:** This is the first report of a *TERT* promoter mutations in HNSCC patients from South America. The high prevalence of *TERT* mutation, as well as its association with poor disease-free survival and overall survival, particular at C228T locus might serve as a prognostic biomarker in HNSCC to help clinicians in the management of treatment.

## Introduction

Approximately 834,860 new cases of head and neck cancer are diagnosed each year in the world that encompasses tumors of the oral cavity, pharynx, and larynx (1). The most common type is squamous cell carcinoma (HNSCC) which accounts for over 90% of all head and neck cancers (2). Usually associated to tobacco and alcohol consumption (3), over the past decades, human papillomaviruses (HPV) have emerged as an important etiological factor for a subset of HNSCC from the oropharynx (4, 5). Despite significant progress in all therapeutic modalities, the 5-year overall survival (OS) rate for HNSCC patients is ~50% and the main reason for treatment failure is the frequent development of loco-regional recurrences (6).

Most HNSCC treatments are associated with high morbidity and toxicity, where recurrent and metastatic disease is usually incurable, highlighting the need for more effective therapies for these patients (7). No new targeted therapies have been approved for HNSCC for decades, other than cetuximab in 2006, which affords only modest response rates (10–15%) as monotherapy (8, 9). The landscape of HNSCC explains the limited response rates of targeted therapies, as most tumors have multiple genetic factors of oncogenesis and are constantly evolving when it comes to therapy (7, 10, 11). In an era of personalized cancer therapy, several investigations are currently examining new biological markers as prognostic and predictive factors in HNSCC (12).

Cancer cells, including HNSCC, are characterized by increased telomerase activity (13). This enzymatic complex is active in ~80–90% of all cancer types and is responsible for the lengthening of telomeres (13, 14). One cancer hallmark is to avoid senescence and unrestricted proliferation, a process called immortalization, and one way to achieve this is by reactivating telomerase in somatic cells (15, 16). Telomerase activity has been consistently detected in 80–90% of malignant tumors (16). Mutations within the promoter region of telomerase reverse transcriptase (*TERT*) that confer enhanced *TERT* promoter activity, have been reported in two major hotspots, which are located at −124 and −146 base pairs upstream of the transcriptional start site (also designated C228T and C250T, respectively) (17–19).

*TERT* promoter mutation has been extensively evaluated in different tumors: thyroid, glioblastoma, urothelial, melanoma, among others (20). Literature reports the use of *TERT* promoter mutation screening programs in thyroid tumors in order to select patients who would benefit from adjuvant treatment and closer follow-up (21), since many studies related the presence of these mutations with poor prognosis (22–25). Glioblastomas also are reported to present a poor prognosis in patients harboring *TERT* mutations, which were commonly evaluated in combination with *IDH* and *MGMT* methylation (26–29). Urothelial carcinoma (30, 31) and melanoma also showed worse prognosis in patients harboring *TERT* mutation (32, 33).

*TERT* promoter mutations resulting in increased telomerase expression have been detected in a significant proportion of HNSCC patients (13, 18, 19, 34–39). It may vary from 16 to 70% of all head and neck subsites, being frequently reported as highly mutated in the oral cavity (37–40). Studies evaluating *TERT* promoter mutation in head and neck patients were only performed in a few countries (United States, China, India, Taiwan, Italy, and Poland). To date, no studies have evaluated these mutations in the Brazilian population, therefore the aim of the present study was to evaluate the prevalence of *TERT* promoter mutations in head and neck cancer patients in Brazil and evaluate for associations with outcome.

## Materials and Methods

### Patient Samples and DNA Isolation

This retrospective study included formalin-fixed paraffin-embedded (FFPE) HNSCC samples from 88 patients surgically treated between 2006 and 2011 at the Department of Head and Neck Surgery of the Barretos Cancer Hospital, Barretos, SP, Brazil. The inclusion criteria were as follows: previously untreated patients with primary HNSCC, submitted to surgery as the first therapeutic modality with curative intent. The use of these samples was approved by the Barretos Cancer Hospital Institutional Review Board. Hematoxylin and eosin sections corresponding to paraffin blocks containing the samples of interest were reviewed by an expert pathologist to confirm the diagnosis and for characterization of the cellular components present in the samples. Scrapings from the region of tissue identified as having at least 80% of tumor cells were processed using QIAamp DNA FFPE Tissue Kit (*Qiagen*, Germany). DNA was quantified in the NanoDrop 2000C (*Thermo Scientific*™) and stored at −20°C until use.

### *TERT* Promoter Mutational Analysis

A pyrosequencing assay was performed to examine these two *TERT* promoter mutations. The primer for pyrosequencing was designed immediately upstream of C250T so that these two mutations are analyzed in the same assay by producing a 162 bp amplicon, which contained the sites of C228T and C250T mutations, as previously described (41). Pyrosequencing assays were performed on a PyroMark Q96ID system using PyroMark Gold reagents (*Qiagen*).

Sanger sequencing was then performed to confirm the results of pyrosequencing. A fragment of the *TERT* promoter region was amplified by PCR using the primers 5′-AGTGGATTCGCGGGCACAGA-3′ and 5′-CAGCGCTGCCTGAAACTC-3′, resulting in a PCR product of 235 bp, which contained the sites of the c.-124 C>T and c.-146 C>T mutations as previously described (26, 42, 43). PCR was performed with initial denaturation at 95°C for 15 min, followed by 40 cycles of denaturation at 95°C for 30 s, annealing at 64°C for 90 s, elongation at 72°C for 30 s, and final elongation at 72°C for 7 min. The quality of PCR products was confirmed by gel electrophoresis. DNA sequencing of the PCR product was performed using the BigDye Terminator version 3.1 Cycle Sequencing Kit (*Applied Biosystems*, USA) and ABI PRISM 3500xL Genetic Analyzer (*Applied Biosystems*, USA). The sequencing reaction was performed in forward direction. An independent PCR amplification/sequencing, in forward direction, was performed in positive samples or samples that were inconclusive.

### Statistical Analysis

Statistical analysis was performed using the software IBM SPSS Statistics 23 for Windows. Categorical variables were compared using Fisher's exact test. Survival curves were calculated by Kaplan–Meier method and differences between groups were compared using the log-rank test. For all analysis, we considered statistical significance when *p* ≤ 0.05.

## Results

### Patient Characteristics

Clinical and histopathological data of the 88 HNSCC patients enrolled in this study are presented in Table 1. Most of the patients profiled in this cohort were male (84.1%) with age ranging from 32 to 82 years (median = 58 years). Tobacco and alcohol consumption were self-reported by 85.9 and 74.4% of the cases, respectively. Tumor sites were subdivided into oral cavity (78.4%), larynx (12.5%), and pharynx (9.1%). Regarding tumor sub-sites within the oral cavity were as follows: 39.8% (35/88) in the oral tongue, 21.6% (19/88) in the floor of the mouth, 12.5% (11/88) in the gums and 4.5% (4/88) in the hard palate and jugal mucosa. Clinical stage was T1/T2 in 27 cases (30.7%) and T3/T4 in 61 (69.3%); 47 (53.4%) of the cases had clinically positive cervical lymph nodes and 41 cases (46.6%) were N0; collectively, 73 (83.0) had advanced disease at diagnosis. Perineural invasion, vascular invasion, and extranodal extension were present in 27 (36.0%), 19 (26.8%), and 23 (32.4%) cases, respectively (Table 1). It was described a self-reported measure of tobacco and alcohol consumption in three categories: yes, no, and former, which were acquired from the patient's medical records. Analysis was performed considering “yes” vs. “no,” where “yes” comprehended smokers or alcohol consumers added to former.

**Table 1:** Clinical and pathological data of the patients enrolled in the study.

**Variable**	**Categories**	***n* (%)**
Age	≤ 60 years	54 (61.4)
	>60 years	34 (38.6)
Gender	Male	74 (84.1)
	Female	14 (15.9)
Tobacco use	Yes	73 (85.9)
	No	12 (14.1)
Alcohol use	Yes	61 (74.4)
	No	21 (25.6)
Anatomic site	Tongue	35 (39.8)
	Floor of mouth	19 (21.6)
	Pharynx	8 (9.1)
	Larynx	11 (12.5)
	Gingiva	11 (12.5)
	Hard palate and jugal mucosa	4 (4.5)
*T*	cT1–cT2	27 (30.7)
	cT3–cT4	61 (69.3)
*N*	cN0	41 (46.6)
	cN+	47 (53.4)
Clinical stage	I/II	15 (17.0)
	III/IV	73 (83.0)
Radiotherapy	Yes	71 (68.3)
	No	33 (31.7)
Chemotherapy	Yes	33 (33.0)
	No	67 (67.0)
Surgical margins	Negative	75 (86.2)
	Positive	12 (13.8)
Extranodal extension	Negative (N0)	33 (46.5)
	No (N+)	15 (21.1)
	Yes (N+)	23 (32.4)
Perineural invasion	Yes	27 (36.0)
	No	48 (64.0)
Vascular invasion	Yes	19 (26.8)
	No	52 (73.2)

### *TERT* Promoter Mutation in Head and Neck Squamous Cell Carcinoma

**Figure 1:**
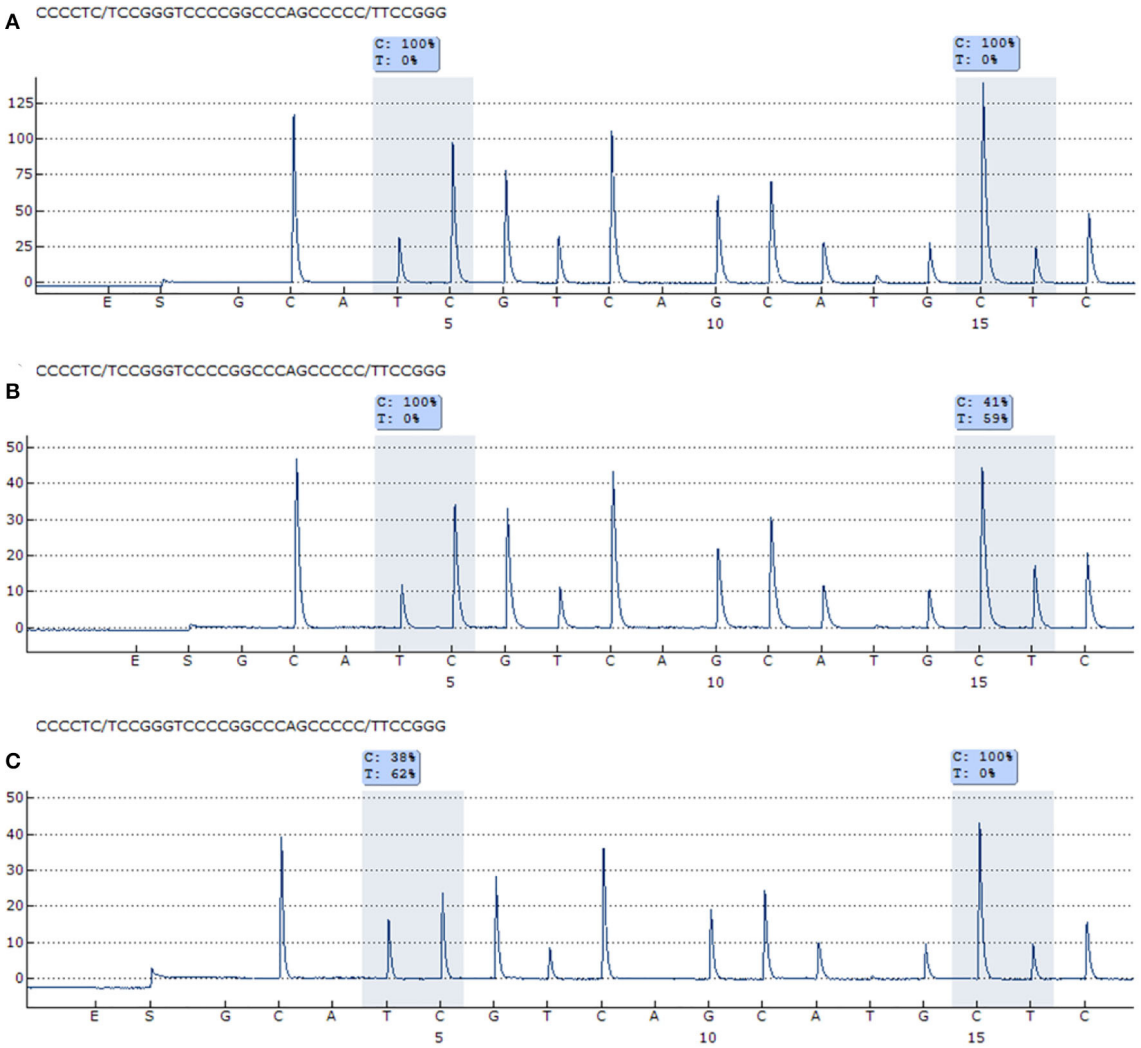
Representative pyrogram of the *TERT* promoter mutations. **(A)** Wild-type *TERT* promoter mutation for C228T and C250T. **(B)** C228T *TERT* promoter mutation (59% of T allele). **(C)** C250T *TERT* promoter mutation (62% of T allele).

To determine the prevalence of *TERT* promoter mutations in this cohort of Brazilian patients with HNSCC, genomic DNA was extracted and pyrosequenced. Primers were used to amplify and sequenced a region containing two previously described recurrent *TERT* promoter mutations (C228T and C250T, Figure 1). The results showed a frequency of 27.3% (24/88) *TERT* mutations in HNSCC, being 20.5% at C250T and 6.8% at C228T hotspot regions (Table 2). The mutations occurred in a mutually exclusive manner with a heterozygous genotype. The mutation frequency of C228T in tongue was 50.0%, in the gums, hard palate and jugal mucosa and pharynx was 16.67%, while the floor of the mouth and larynx did not present this mutation (Table 2). Regarding C250T mutation, the frequency was 55.56% for tongue, 33.33% for the floor of the mouth, 5.56% for hard palate and jugal mucosa, and for larynx, while the gums and the pharynx did not present this mutation (Table 2). When HNSCC sub-sites were grouped in oral cavity, pharynx and larynx, *TERT* mutations were present in 31.9, 12.5, and 9.1% of the cases, respectively.

**Table 2:** *TERT* promoter mutation in HNSCC samples according to tumor sub-sites.

**Tumor sub-site**	**C228T *n* (%)**	**C250T *n* (%)**	**WT *n* (%)**
Tongue	3 (50.0)	10 (55.56)	22 (34.38)
Floor of the mouth	0 (0.0)	6 (33.33)	13 (20.13)
Gums	1 (16.67)	0 (0.0)	10 (15.63)
Hard palate and jugal mucosa	1 (16.67)	1 (5.56)	2 (3.13)
Larynx	0 (0.0)	1 (5.56)	10 (15.63)
Hypopharynx	1 (16.67)	0 (0.0)	1 (1.56)
Oropharynx	0 (0.0)	0 (0.0)	6 (9.37)
Total	6 (6.8)	18 (20.5)	64 (72.7)

### *TERT* Promoter Mutation Correlation With Clinical and Pathological Features

In a univariate analysis, 94.4% of the patients harboring *TERT* promoter mutation C250T were alcohol consumers (*p* = 0.032). Moreover, 66.7% of the patients harboring *TERT* promoter mutation C228T were non-alcohol consumers (*p* = 0.035). When considering cases with either one of the mutations tested, no statistically significant association between the presence of mutation (C228T or C250T vs. wild-type) and clinical-pathological features was observed.

**Figure 2:**
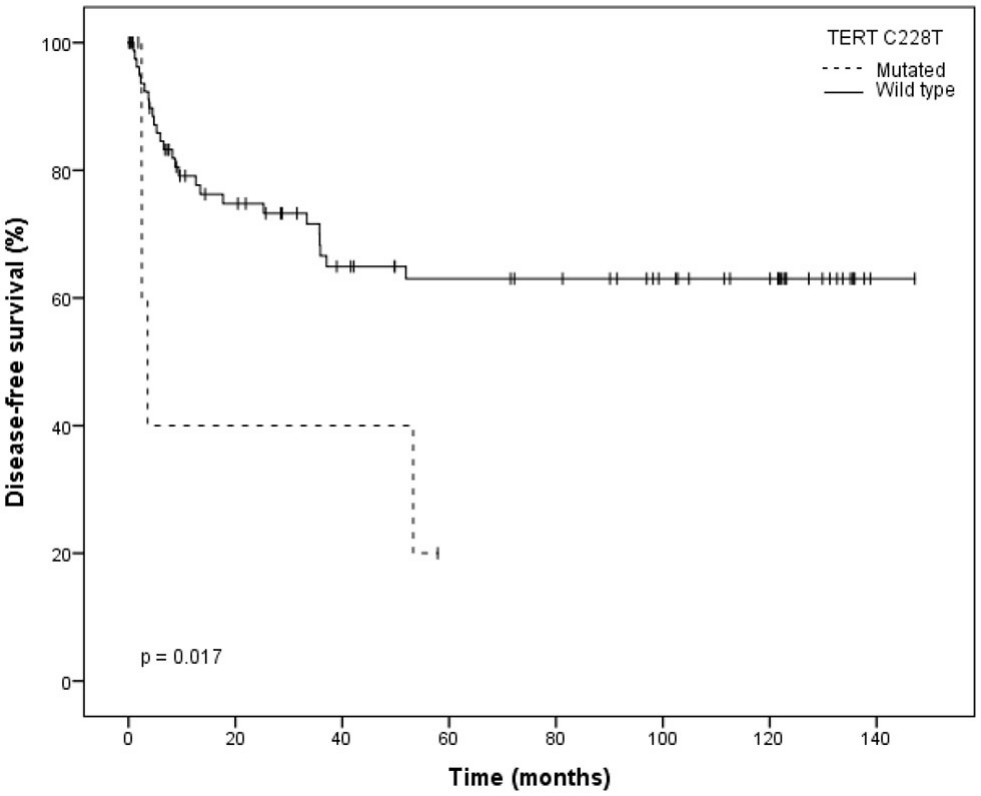
Kaplan–Meier plot for disease-free survival (DFS) stratified by wild type and C228T mutation type.

Kaplan–Meier survival curves were used to estimate survival according to *TERT* promoter mutation status. No statistically significant association between the presence of mutation (C228T or C250T vs. wild-type) and survival was observed. Also, no statistically significant association between the presence of mutation C250T and survival was observed. Importantly, we observed that the 5-year disease-free survival (DFS) for patients harboring mutation C228T was 20.0 vs. 63.0% for patients without this mutation (*p* = 0.017; Figure 2).

Univariate analysis of the effect of this mutation on DFS of patients showed that C228T *TERT* promoter mutation was significantly associated with an increased risk of tumor relapse (HR = 3.372; 95% CI: 1.17–9.73; *p* = 0.025; Table 3). Lower disease-free survival was associated, as expected, with the following clinical characteristics: N-stage (log-rank *p* = 0.04), extranodal extension (log-rank *p* = 0.031) and surgical margins (log-rank *p* = 0.038; Table 3).

**Table 3:** Results of univariate analysis of selected prognostic factors for disease-free survival.

**Characteristics**	**Number of cases**	**Numberv of recurrent cases**	**5-year disease-free survival**	***P*-value (long-rank)**	**Hazard ratio for recurrence (95% CI)**	***P*-value (Cox)**
**Tobacco use**						
No	12	6	42.4	0.237	Reference	0.243
Yes	73	23	63.7		0.585 (0.238–1.438)	
**Alcohol use**						
No	21	10	44.1	0.052	Reference	0.057
Yes	61	16	69.1		0.464 (0.210–1.025)	
**Clinical tumor status**						
T1–T2	27	10	59.8	0.811	Reference	0.811
T3–T4	61	20	60.5		1.097 (0.513–2.348)	
**Clinical nodal status**						
N0	41	10	70.8	**0.040**	Reference	**0.045**
N+	47	20	50.6		2.177 (1.017–4.660)	
Clinical TNM stage^*^						
Initial (I/II)	15	5	64.3	0.584	Reference	0.585
Advanced (III/IV)	73	25	59.6		1.307 (0.500–3.421)	
**Anatomic site**						
Oral cavity	69	23	61.7	0.937	Reference	0.937
Pharynx and larynx	19	7	52.6		0.967 (0.414–2.256)	
**Surgical margins**						
Negative	75	22	66.0	**0.038**	Reference	**0.044**
Positive	12	7	29.1		2.395 (1.022–5.612)	
**Extranodal extension**						
Negative (N0)	33	6	76.8	0.31	Reference	
No (N+)	15	5	61.4		2.093 (0.639–6.862)	0.223
Yes (N+)	23	11	41.7		3.560 (1.312–9.660)	**0.013**
**Perineural invasion**						
No	48	13	68.1	0.105	Reference	0.111
Yes	27	12	49.7		1.895 (0.864–4.158)	
**Vascular invasion**						
No	52	17	62.9	0.357	Reference	0.361
Yes	19	8	50.8		1.483 (0.637–3.451)	
**C228T mutation**						
No	82	26	63.0	**0.017**	Reference	**0.025**
Yes	6	4	20.0		3.372 (1.169–9.730)	
**C250T mutation**						
No	70	24	59.6	0.992	Reference	0.992
Yes	18	6	62.8		1.005 (0.410–2.461)	
**C228T or C250T mutation**						
No	64	20	63.3	0.250	Reference	0.254
Yes	24	10	51.2		1.557 (0.728–3.331)	

* *Clinical stage according to TNM Classification of Malignant Tumors−7th ed. Bold values indicates the statistical significance (p ≤ 0.05)*.

**Figure 3:**
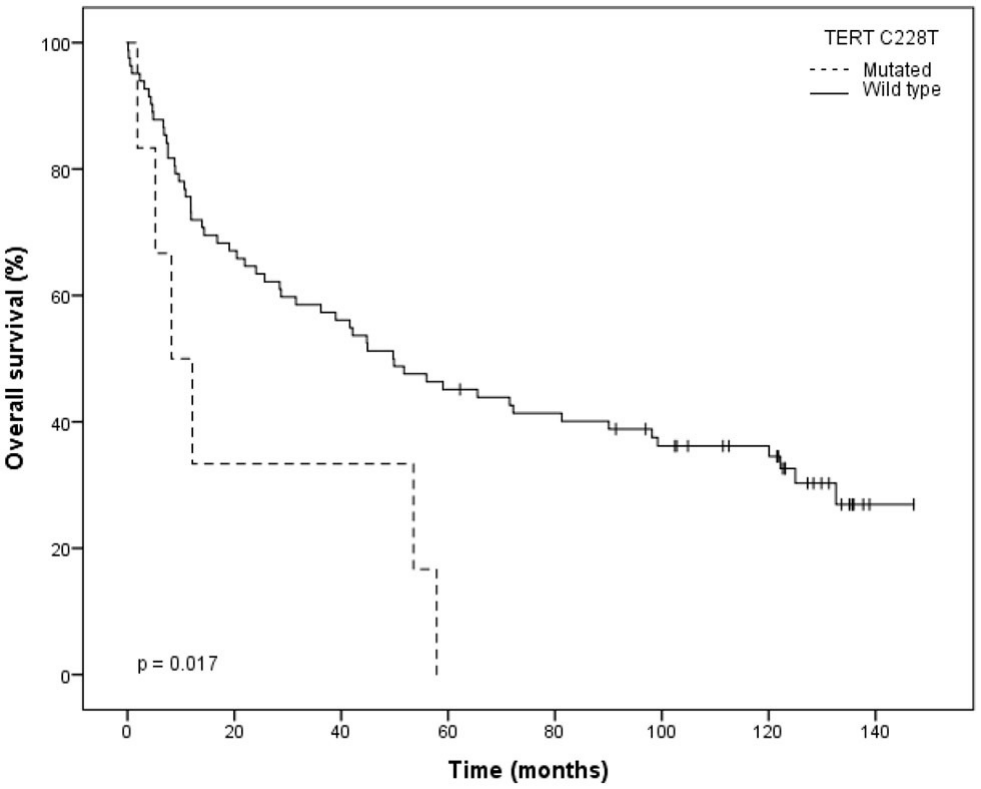
Kaplan–Meier plot for overall survival (OS) stratified by wild type and C228T mutation type.

The same negative impact of C228T *TERT* promoter mutation was observed in the 5-year overall survival (OS) with only 16.7% of the cases found alive after 5 years, in comparison to 45.1% of patients without this mutation (*p* = 0.017; Figure 3).

Also, a statistically significant increased risk of death was also observed for the cases harboring this C228T mutation (HR = 2.708; 95% CI: 1.15–6.374; *p* = 0.023; Table 4). A decrease in overall survival was also associated with important clinical factors such as: N-stage (log-rank *p* = 0.020), T-stage (log-rank *p* = 0.031), clinical stage (log-rank *p* = 0.018), surgical margins (log-rank *p* = 0.031) and perineural invasion (log-rank *p* = 0.032; Table 4).

**Table 4:** Results of univariate analysis of selected prognostic factors for overall survival.

**Characteristics**	**Number of cases**	**Number of deaths**	**5-year overall survival**	***P*-value (long-rank)**	**Hazard ratio for death (95% CI)**	***P*-value (Cox)**
**Tobacco use**						
No	12	8	50.0	0.924	Reference	0.924
Yes	73	51	41.1		1.037 (0.492–2.186)	
**Alcohol use**						
No	21	16	38.1	0.343	Reference	0.345
Yes	61	40	45.9		0.756 (0.423–1.351)	
**Clinical tumor status**						
T1–T2	27	16	63.0	**0.031**	Reference	**0.034**
T3–T4	61	46	32.8		1.858 (1.049–3.291)	
**Clinical nodal status**						
N0	41	24	53.7	**0.020**	Reference	**0.022**
N+	47	38	31.9		1.819 (1.089–3.038)	
Clinical TNM stage^*^						
Initial (I/II)	15	7	80.0	**0.018**	Reference	**0.022**
Advanced (III/IV)	73	55	34.2		2.513 (1.140–5.537)	
**Anatomic site**						
Oral cavity	69	48	44.9	0.905	Reference	0.905
Pharynx and larynx	19	14	31.6		1.037 (0.570–1.886)	
**Surgical margins**						
Negative	75	49	46.7	**0.031**	Reference	**0.034**
Positive	12	12	16.7		2.001 (1.054–3.799)	
**Extranodal extension**						
Negative (N0)	33	20	51.5	0.202	Reference	
No (N+)	15	11	33.3		1.380 (0.660–2.882)	0.392
Yes (N+)	23	18	30.4		1.777 (0.938–3.367)	0.078
**Perineural invasion**						
No	48	28	52.1	**0.032**	Reference	**0.035**
Yes	27	23	33.3		1.818 (1.043–3.169)	
**Vascular invasion**						
No	52	32	48.1	0.088	Reference	0.092
Yes	19	16	36.8		1.681 (0.919–3.073)	
**C228T mutation**						
No	82	42	16.7	**0.003**	Reference	**0.023**
Yes	6	6	52.9		2.708 (1.150–6.374)	
**C250T mutation**						
No	70	40	48.1	0.606	Reference	0.733
Yes	18	8	53.6		0.893 (0.465–1.715)	
**C228T or C250T mutation**						
No	64	45	46.9	0.445	Reference	0.446
Yes	24	17	29.2		1.243 (0.710–2.177)	

* *Clinical stage according to TNM Classification of Malignant Tumors−7th ed. Bold values indicates the statistical significance (p ≤ 0.05)*.

Finally, a multivariable Cox regression model including alcohol consumption, N-stage, surgical margins and the status of C228T for disease-free survival was performed and, only the status of C228T remained significant (HR = 3.372; 95% CI: 1.169–9.730; *p* = 0.025). In the multivariable Cox regression model including T-stage, N-stage, clinical stage, surgical margins, and the status of C228T for overall survival, clinical stage remained significant (HR = 2.373; 95% CI: 1.072–5.256; *p* = 0.033) and the status of C228T was marginally significant (HR = 2.352; 95% CI: 0.995–5.558; *p* = 0.051).

## Discussion

*TERT* expression is downregulated as a normal cell divides, resulting in telomere shortening and replicative senescence (36, 44). Telomere length is important for cell cycle regulation, cell senescence, and genetic instability regulation (45). In most cancers, *TERT* expression is reactivated and overexpressed during tumorigenesis leading to replicative immortality (35, 44).

*TERT* promoter mutation has been heavily reported in melanoma, glioma, urothelial, thyroid, hepatocellular, and non-small cell lung cancer (17, 19). For head and neck tumors, the frequency of those mutations varies significantly among studies (Table 5). These differences could be explained by tumor sub-site, sample size, methodological sensitivity, risk factors, and population ethnicity. In our cohort, 27.5 of cases showed *TERT* promoter mutation, being higher in the C250T than in the C228T locus, 20.5 and 6.8%, respectively. Inversely to those results, when all sites in the head and neck were considered, *TERT* promoter mutations of C250T and C228T were observed in 2.8 and 14.8% in Killela et al. (19) study, 0 and 16.6% in Cheng et al. (35) study and 6.3 and 30.2% in Morris et al. (39) study, respectively (Table 5). When only the oral cavity is considered, *TERT* promoter mutations of C250T was also more frequent than C228T in our study, 24.6 and 7.2%, respectively, corroborating to Barczak et al. (13) report of 40.0% of C250T mutation being in the mouth. Conversely to our results, frequency of C250T and C228T were 9.7 and 22.0% in Vinothkumar et al. (36) study, 12.9 and 51.7% in Chang et al. (37) study, 13.3 and 15% in Annunziata et al. (38) study and 20 and 55% in Morris et al. (39) study, respectively (Table 5). In Boscolo-Rizzo et al. (40) study, both mutations were evaluated without distinction, showing 83.3% frequency in oral cavity. For larynx, our results showed 5.5% of C250T and 0% of C228T mutation. Similarly, Qu et al. (34) reported 23.8% frequency for mutation C250T and 3.4% for C228T, which also differs from Morris et al. (39) laryngeal cohort, reported to be 0% in C250T and 14.3% in C228T (Table 5). Those differences could be explained due to the different proportion of head and neck sub-sites, the number of samples analyzed, and the ethnicity of the patient population (USA, China, India, Taiwan, Poland, and Italy). Brazilian head and neck cancer patients are more likely to be heavy tobacco and alcohol consumers (3) than HPV^+^ (46–48), which can explain the mutagenic effect on the mucosal epithelia of the upper aerodigestive tract. At Barretos Cancer Hospital, we use p16-immunohistochemistry (p16-IHC) as a surrogate marker for HPV infection, as recommended by the 8th edition of AJCC TNM staging system specifically for oropharyngeal squamous cell carcinomas. For the 6 oropharynx SCC evaluated in this study (all WT *TERT*), p16-IHC was only available for four of them: 1/4 was classified as p16-positive and 3/4 as HPV-negative. Further studies are required in order to assess the correlation between *TERT* and HPV.

**Table 5:** Summary of studies evaluating the association between head and neck tumors with *TERT* promoter mutations.

**Study**	**Tumor Site**	**Total *TERT* promoter mutation % (n)**	**C228T % (*n*)**	**C250T % (*n*)**	**Results**	**Method**	**Country**
(19)	Head and neck	17.1 (12/70)	14.8 (10/70)	2.8 (21/70)	*TERT* mutations were correlated to tongue sub-site (*p* < 0.0001)	PCR and Sanger sequencing	USA
(34)	Larynx	27.0 (64/235)	3.4 (8/235)	23.8 (56/235)	Laryngeal *TERT* mutations were significantly associated with poor survival (*p* = 0.03). The cases with *TERT* promoter mutations had significantly shorter survival than those with wild-type *TERT* (72.2 vs. 78.2 months, *p* = 0.04). Also, *TERT* C250T mutation was significantly associated with worse survival (69.2 vs. 78.2 months, *p* = 0.01)	Pyrosequencing	China
(35)	Head and neck	16.6 (2/12)	16.6 (2/12)	0.0 (0/12)	No significant correlation was observed	PCR and Sanger sequencing	USA
(36)	Oral cavity	31.7 (13/41)	22.0 (9/13)	9.7 (4/13)	No significant correlation was observed	PCR and Sanger sequencing	India
(37)	Oral cavity	64.7 (130/201)	51.7 (104/201)	12.9 (26/201)	The C228T mutation in oral cavity was associated with betel nut chewing (*p* = 0.05)	PCR and Sanger sequencing	Taiwan
(13)	Head and neck	63.93 (39/61)		63.93 (39/61)	The C250T mutation in HNSCC indicated an association between the frequency of the homozygous mutation (T/T) and the grade of the tumor (*T*1 = 27%; *T*2 = 36%; *T*3 = 35%; *T*4 = 46%; *P* ≤ 0.0001). Also, C250T mutation was identified in 40% of patients with mouth cancer (*P* = 0.001)	High resolution melting using qPCR	Poland
(18)	Head and neck	28.6 (8/28)			No significant correlation was observed	Next-generation sequencing	USA
(38)	Oral Cavity	60.0 (9/15)	20.0 (3/15)	13.3 (2/15)	No significant correlation was observed	PCR and Sanger sequencing	Italy
	Oropharynx	0.0 (0/15)	0.0 (0/15)	0.0 (0/15)			
(39)	Oral Cavity	70.0 (14/20)	55.0 (11/20)	15.0 (3/20)	Frequency of *TERT* promoter mutation was higher in the metastasis than in the primary tumors (*p* < 0.0001)	MSK-IMPACT assay next-generation sequencing	USA
	Oropharynx	5.5 (1/18)	5.5 (1/18)	0.0 (0/18)			
	Hypopharynx	0.0 (0/2)	0.0 (0/2)	0.0 (0/2)			
	Larynx	14.3 (1/7)	14.3 (1/7)	0.0 (0/7)			
(40)	Oral Cavity Pharynx Larynx	9.90 (10/101) 0.99 (1/101) 0.99 (1/101)			No significant difference in OS emerged for mutational status, although 5-year OS was higher in patients with TERT mutations than in those without mutations. TERT levels were not associated with TERT mutations	PCR and Sanger sequencing	Italy
Present study	Oral Cavity Larynx Pharynx	31.9 (22/69) 5.6 (1/11) 16.7 (1/8)	7.2 (5/69) 0 (0/11) 16.7 (1/8)	24.6 (17/69) 5.6 (1/11) 0 (0/8)	C250T *TERT* promoter mutation was associated with alcohol consumption (*p* = 0.032), whereas C228T was not associated with alcohol consumption (*p* = 0.035). The presence of C228T mutation impacted patient outcome, with a significant decrease in disease-free survival (20.0 vs. 63.0%, *p* = 0.017) and in overall survival (16.7 vs. 45.1%, *p* = 0.017)	Pyrosequencing	Brazil

Our data showed a trend in significance with 91.7% of all mutations occurring in the oral cavity (*p* = 0.068), which was similar to the results reported by Barczak et al. (13) and Killela et al. (19) stating that *TERT* mutation was correlated to oral cavity, most specifically tongue sub-site.

Interesting, a comparison study evaluating *TERT* mutation in a head and neck metastatic cohort, the mutation was significantly more frequent in the recurrence then in the primary HNSCC tumors (39). Unfortunately, we did not have the metastatic tumor to compare its mutation profile with the primary tumor in our cohort. Moreover, Chang et al. (37) reported that *TERT* mutation C228T in oral cavity was also correlated to betel nut chewing, while our study did not find association with tobacco consumption. In contrast, this report found that 94.4% of the patients harboring *TERT* promoter mutation C250T were alcohol consumers while 66.7% of the patients harboring C228T were non-alcohol consumers, in a cohort with 74.4% of alcohol consumers patients.

Importantly, the present data was able to show the effect of *TERT* mutation C228T on the 5-year disease-free survival, which was 20.0% for patients harboring this mutation vs. 63.0% for patients without this mutation, being therefore associated with an increased risk of tumor relapse. The same negative impact of *TERT* promoter mutation C228T was observed in the 5-year overall survival with only 16.7% of the cases found alive after 5 years, in comparison to 45.1% of patients without this mutation, also associated with a statistically significant increased risk of death. Corroborating our results, Qu et al. (34) found *TERT* mutation associated with poor overall survival in laryngeal tumor patients (cases with *TERT* promoter mutations had 72.2 vs. 78.2 months for wild-type patients, *p* = 0.04). The mechanism by which *TERT* promoter mutations ultimately facilitate cancer progression and can constitute prognostic factors are not fully elucidated. It has been reported that C228T and C250T mutations are functionally distinct, with C228T leading to GABPA recruitment, whereas C250T generate both an ETS site and a functional p52 site requiring ETS1/2 (49). In the present study, we identified only C228T mutations association of worse outcome. This could be due to small size of patients C228T mutated and further studies with a large number should be done to extend and validate this finding.

Chiba et al. (50) recently discovered that *TERT* promoter mutations may occur in two phases: (1) early stages of tumorigenesis resulting in telomerase activity, which is insufficient to prevent telomere shortening and (2) during subsequent divisions, when the number of short telomeres increases, and telomerase activity becomes rate-limiting, causing telomere-driven genomic instability. Therefore, those mutations promote a dual role in tumorigenesis: cancer cell immortalization and genome instability (50), which seem to be responsible for the increased risk of tumor relapse and increased risk of death in patients harboring the mutation C228T.

Recent studies have reported the feasibility of TERT promoter mutation detection and its applicability in screening programs for patients with thyroid cancer to predict patient's outcome (21, 23). Our study suggests that in head and neck tumors, TERT promoter mutations could be performed in a screening setting to help clinicians decide for a more aggressive initial treatment accompanied by a closer follow-up, in order to give these patients a better chance to survive. Studies validating these results should be performed in order to confirm the predictive value of this marker for prognosis. In addition, minimally invasive approaches to detect TERT promoter mutations could be performed in head and neck samples in order to evaluate the feasibility of this marker, and since oral cavity is the most affected site, saliva could possibly be a good source, taking into consideration previous data in other tumors: FNA (Fine-Needle Aspiration) in thyroid tumors (51–53) and cell-free DNA (cfDNA) from urine in urothelial tumors (54–56), which were able to detect the mutation in fluids.

In conclusion, to the best of our knowledge, this is the first study evaluating Brazilian population reporting high prevalence of *TERT* promoter mutations in HNSCC and associating with poor disease-free survival and overall survival. Thus, *TERT* promoter mutation C228T might serve as a prognostic biomarker in head and neck squamous cell carcinoma to help clinicians in the management of treatment.

## Data Availability Statement

The datasets generated for this study are available on request to the corresponding author.

## Ethics Statement

The studies involving human participants and use of these samples were reviewed and approved by the Barretos Cancer Hospital Institutional Review Board. The patients/participants provided their written informed consent to participate in this study.

## Author Contributions

RR, CS-N, and ALC designed the study. LA conducted the study, performed the data analysis and interpretation, statistical analysis, and wrote the manuscript. AC-C conducted the study and performed the data analysis. ACC and BS performed the data interpretation and statistical analysis. BS compiled the clinical data. All authors contributed to the article and approved the submitted version.

## Conflict of Interest

The authors declare that the research was conducted in the absence of any commercial or financial relationships that could be construed as a potential conflict of interest.
